# Analyzing the Dimensions of the Quality of Life in Hepatitis B Patientsusing Confirmatory Factor Analysis

**DOI:** 10.5539/gjhs.v7n7p22

**Published:** 2015-03-26

**Authors:** Ghassem Abedi, Farideh Rostami, Aliasghar Nadi

**Affiliations:** 1 Health Sciences Research Center, Mazandaran University of Medical Sciences, Sari, Iran

**Keywords:** chronic liver disease questionnaire, confirmatory factor analysis, Hepatitis B, Quality of Life, Mazandaran, WHOQOL-BREF

## Abstract

**Background and Objectives:**

The scope of the quality of life assessment is not widespread in any time like today. Economists, social scientists and politicians look at this topic from the particular approach. The life quality in hepatitis B patients regarding the degree of its progress is considered a major concern in these patients. Thus, the aim of the study was analyzing the dimensions of the life quality of a group of people suffering from hepatitis B in Mazandaran province in 2012.

**Methods:**

This study was done by descriptive, cross-sectional method on 210 (118 women and 92 men) hepatitis B patients that six month have passed from their diagnosis and formation of follow-up form in health centers, using access sampling method atsix regions of Mazandaran province. The instruments of the study were the questionnaire of World Health Organization questionnaire (WHOQOL-BREF) and the chronic liver disease questionnaire (CLDQ). For analyzing the data from descriptive statistics and Kolmogrov-Smirnov test, one sample t-test, two stage Confirmatoryfactor analysis, Spss and Lisrel software has been used.

**Results:**

Findings showed that the social relationship dimension with factor loading of 0.81 has the most amount of coefficient of effectiveness; physical health with factor loading of 0.72, mental health with factor loading of 0.63 and environmental health with factor loading of 0.55 have the least amount of coefficient of effectiveness in creating the generallife quality of hepatitis B patients. In the quality of life in hepatitis patients, the emotional function with factor loading of 0.76 has the most coefficient of effectiveness, activity dimension with factor loading of 0.67, fatigue withfactor loading of 0.47, abdominal syptoms with factor loading of 0.42 and worry with factor loading of 0.32 have the least coeficient of effectivness in making CLDQ domains of hepatit B patients.

**Conclusions:**

The general quality of life in patients had been below average and social relationship and emotional function must be properly investigated and managed in hepatitis B patients in order to improve life quality. WHOQOL-BREF and CLQD proved to be a useful instrument to assess general life quality in patients and can be helpful to find practical strategies to improving life quality in these patients.

## 1. Background

The scope of the quality of life assessment is not widespread in any time like today. Economists, social scientists and politicians look at this topic from the particular approach (Bondini et al., 2007; Strauss et al., 2006; Merat et al., 2004). The indicators of life quality include the large range from food and clothing to health care and social- physical environment (Sobhonslidsuk et al., 2006). Although the life quality has been translated to life level in some resources, but life level and material development includes only one of the basics of life quality (Awan, Waqas, & Aslam, 2011). In fact, the concept of life quality is a composite variable that is influenced by several variables (Sharif, Mohebbi, Tabatabaee, Saberi-Firoozi, & Gholamzadeh, 2005). Despite different definitions of life quality, there has not been a consensus regarding the definition to enfold the various aspects of this concept. The World Health Organization (WHO) defines quality of life as; “Individuals’ perception of their position in life in the context of the culture and value systems in which they live and in relation to their goals, expectations, standards and concernsquality (Awan, Waqas, & Aslam, 2011). Currently, the scope of life quality and its assessment in chronic diseases have been studied widely. In chronic disease, the main purpose of health care monitoring and treatment is life satisfaction and wellbeing feeling. However, the life quality of patients with chronic hepatitis B is often below the normal range (Kramer et al., 2005). Studies showed that with the progression of liver disease and ineffective anti-viral treatment, the physical and mental health of patients damage increasingly (Bjornsson et al., 2009; Kanwal et al., 2005). These patients suffer from fatigue, loss of confidence, inability to work, anxiety, depression and other emotional problems that reduce severely their life quality (Pojoga et al., 2004). According to results from previous studies and agreement about the reducing of life quality with regards to disease progression (Alavian et al., 2008; Nokhodian et al., 2009), however, in this study in terms of the cultures and value systems, the life situations have different goals, expectations, standards and priorities that is not clear with others. Perhaps research about the life quality at the group of patients in different situations leads to modern steps to compare with mathematical techniques to solve medicine problems and other problems. Thus, the aim of this study was analyzing the dimension of quality of life in hepatitis B using Confirmatory factor analysis (CFA) in the Mazandaran province.

## 2. Methods

The study was conducted in descriptive cross sectional form on 210 persons (110 women and 92 men) hepatitis B patients that six month have passed from their diagnosis and formation of follow-up form in health centers, in Sari, Neka, Qaemshahr, Amol, Nur, and Tonekabon in Mazandaran province using access method among patients more than 18 years old in 2012. The data collection method was based on two questionnaires; WHO questionnaire (WHOQOL-BREF) for measuring generallife quality include 24 questions about dimensions such as; physical health, mental health, social relations and environmental health and quality of life index for patients with chronic liver disease (CLDQ) with some reduction and changes in questionnaire’ dimension, that included questions about abdominal symptoms, activity, fatigue, emotional function and worry. This study was done with the Confirmatory Factor Analysis (CFA) method. On the basis of the two special features, such analyses i.e. estimating the “standard factor loading” and measuring the “model fitting” are used in desirability analysis. In this study, after model fitting based on standard coefficient we dealt with ranking every one of the constituents of generallife quality. In this research at first the main domains of generallife quality including physical health, psychological health, social relationship, and environmental health and then in each of the health components their constitute elements in the model are also considered. Thus, in examining the quality of life index for patients with chronic liver disease in the main domain we dealt with examining the constituents of the quality of life in hepatitis patients like abdominal symptoms, activity, fatigue, emotional symptoms and concern and in the itemsin each of the components of the constituents elements are also considered. For describing data central indices, for examining the present state of each variable, one sample t-test and for determining the current statusvariables of generallife qualityand (CLDQ), two Stage Confirmatory factor analysis (CFA) and standard factor loading and t-values have been used. For examining the adequacy of model, chi-square indices, Root Mean Square Error of Approximation (RMSEA), Normed Fit Index (NFI), Non-Normed Fit Index (NNFI), Root Mean Square Residual (RMR), Goodness of Fit Index (GFI), Adjusted Goodness of Fit Index (AGFI) and Incremental Fit Index (IFI) have been used.

## 3. Results

Since the questionnaire has Likert with five options, and the 3 represents theaverage value, sowe use one sample t- test with test value 3 to study the current status of general life quality and CLDQ (Table 1).

**Table 1:** QoL and CLDQ domains in hepatitis B patients as one sample t-test

variables	Domains	Test value=3				

		Mean	Standard deviation	t	d.f	Sig.
**General life quality**	Physical health	2.79	0.76	-3.88	209	<0.001
	Mental health	2.81	0.90	-2.89	209	<0.01
	Social relationship	2.80	0.76	-3.75	209	<0.001
	Environmental health	2.74	0.72	-5.06	209	<0.001
**CLDQ**	Abdominal symptoms	2.20	1.21	-9.52	209	<0.001
	Activity	2.54	1.20	-5.48	209	<0.001
	Fatigue	2.22	1.15	-9.76	209	<0.001
	Emotional function	2.64	1.01	-5.10	209	<0.001
	Worry	2.36	1.09	-8.39	209	<0.001

As you see in Table 2, results of confirmatory factor analysis and determining of amount of factor loading of all identified general life quality components:social relationship dimension with factor loading 99% involves the highest effectiveness coefficient, physical health dimension with factor loading 55%, environmental health dimension with factor loading 57%, mental health dimension with factor loading 37% involves the lowest effectiveness coefficient interfere in creation of the general life quality of patients infected with hepatitis B, in fact, from patient ’s point of view, mental health dimension is the most important dimension and environmental health is the least important dimension of general life quality.

**Table 2:** Factor analysis of general life quality and CLDQ domains in hepatitis B patients

Variable	Dimensions	Standard factor loading	t	R^2^	Result
**General life quality**	Physical health	0.55	6.47	0.31	Sig.
	Mental health	0.37	4.88	0.13	Sig.
	Social relationship	0.99	9.18	0.98	Sig.
	Environmental health	0.57	5.95	0.33	Sig.
**CLDQ**	Abdominal symptoms	0.42	3.94	0.18	Sig.
	Activity	0.67	4.96	0.45	Sig.
	Fatigue	0.47	4.52	0.22	Sig.
	Emotional function	0.76	6.52	0.57	Sig.
	Worry	0.32	3.54	0.10	Sig.

In the meanwhile, all the factor loading with t value more than 2 are significant at the error level of 0.05 (the extent of being significant is that the calculated t absolute value is more than 1.96). Also, they estimate the considerable amount of variance of the relevant elements (the amount of determination coefficient or R^2^ between 31 to 65 percent). Also the results of confirmatory factor analysis and determining the amount of factor loading of every one of the identified components of health were: The dimension of emotional function with factor loading of 0.76 has the most amount of affecting coefficient, the dimension of activity with factor loading of 0.67, the fatigue dimension with factor loading of 0.47, abdominal symptoms dimension with factor loading of 0.42 and worry dimension with factor loading of 0.32 have the least amount of affecting coefficient in creating of CLDQ domains of patients having hepatitis B. In fact, emotional dimension is the most important and anxiety dimension is the least important dimension in clarifying the CLDQ domains from patient’s perspective. In the meanwhile all the factor loading with t-value more than 2 are significant at the error level of 0.05 (the extent of being significant is that the calculated t absolute value is more than 1.96). Also, they estimate the considerable amount of the variance of the relevant elements (the amount of determination coefficient or R^2^ between 10 to 57 percent).

With regard to the Table 3, the results of R^2^ and determining the amount of factor loading each of the components in general health in physical health dimension of the superficial form component with factor loading of 0.97 have the most amount of affecting coefficient and physical ability component with factor loading of 0.46 has the least amount of effect. In the psychological health dimension, the enjoying of life component with the factor loading of 0.99 has the most amounts and anxiety component with factor loading of 0.83 has the least amount of affecting coefficient. In the social relationship component, the relationship with other members of family with factor loading of 0.80 has the most amount and visit component with factor loading of 0.69 has the least amount of affecting coefficient and in the environment health dimension, accessing to the information component with factor loading of 0.81 has the most amount and Access to health services with factor loading 0.41 has least amount of affecting coefficient in clarifying the environment health dimension.

**Table 3:** General life quality in hepatitis patients according todomains andtheir items

Dimensions	Items	Standard factor loading	R^2^	Result	Priority
**Physical health**	Work capacity	0.76	0.58	Sig.	2^nd^
	Enough energy	0.97	0.95	Sig.	1^st^
	Able to go around	0.52	0.27	Sig.	5^th^
	Daily living activity	0.46	0.21	Sig.	7^th^
	Medical treatment	0.48	0.23	Sig.	6^th^
	Physical pain	0.71	0.51	Sig.	4^th^
	Sleep	0.72	0.52	Sig.	3^rd^
**Emotional health**	Enjoy life	0.99	0.98	Sig.	1^st^
	Life be meaningful	0.83	0.68	Sig.	5^th^
	Concentration	0.83	0.69	Sig.	6^th^
	Satisfy with yourself	0.93	0.86	Sig.	2^nd^
	Appearance	0.91	0.83	Sig.	3^rd^
	Feeling blue mood/respire/anxiety/depression	0.85	0.72	Sig.	4^th^
**Social health**	Relationship with other members of family	0.80	0.63	Sig.	1^st^
	Sex relations	0.70	0.49	Sig.	2^nd^
	Agreement with others and self	0.69	0.47	Sig.	3^rd^
**Environmental health**	Security	0.59	0.35	Sig.	6^th^
	Healthy living environment	0.69	0.47	Sig.	3^rd^
	Access to information	0.81	0.66	Sig.	1^st^
	Recreational activities	0.80	0.64	Sig.	2^nd^
	Life location	0.67	0.45	Sig.	4^th^
	Transport	0.64	0.42	Sig.	5^th^
	Access to health services	0.41	0.17	Sig.	8^th^
	Enough money to meet yourself	0.55	0.31	Sig.	7^th^

With regard to the Table 4, the exploratory factor analysis (EFA) results and determining the amount factor loading of each one of the components in CLDQ domainsin abdominal symptoms items; feeling the pain and with factor loading 0.69 have the most amount of affecting coefficient and dysfunctionand treatment with factor loading of 0.68 has the least effect. In activity dimension, fitness and exercise with factor loading of 0.65 have the most and daily walk item with factor loading of 0.54 has the least amount of affecting coefficient. In fatigue dimension, the feeling tiredin sports with factor loading of 0.75 has the most and fatigue atsome timewith factor loading of 0.55 has the least amount of affecting coefficient. At emotional dimension, compassionrather thanaround with factor loading of 0.80has the most and gloom and lonelinesswith factor loading of 0.75 has the least amount of affecting and in worrydimension, stress with factor loading of 0.89 has the most and concern item with factor loading of 0.66 has the least amount of affecting in clarifying every one of CLDQ domains.

**Table 4:** CLDQ domains in hepatitis patients as dominos and their items

Dimensions	Items	Standard factor loading	t	Result	Priority
Abdominal symptoms	Feeling the pain	0.69	0.48	Sig.	1^st^
	Dysfunction	0.68	0.48	Sig.	2^nd^
	Treatment	0.68	0.46	Sig.	2^nd^
	Normal daily activities	0.59	0.35	Sig.	2^nd^
Activity	Daily walk	0.54	0.30	Sig.	3^rd^
	Fitness and Exercise	0.65	0.43	Sig.	1^st^
	Fatigue during the day	0.70	0.49	Sig.	2^nd^
Fatigue	Fatigue when walking	0.68	0.46	Sig.	3^rd^
	Fatigue at sometime	0.55	0.30	Sig.	4^th^
	Feeling tired in Sports	0.75	0.56	Sig.	1^st^
Emotional function	Loneliness	0.75	0.56	Sig.	2^th^
	Compassion rather than around	0.80	0.64	Sig.	1^st^
	Gloom	0.75	0.56	Sig.	2^th^
Worry	Stress	0.89	0.80	Sig.	1^st^
	Depressions	0.71	0.51	Sig.	5^th^
	Anxiety	0.73	0.54	Sig.	4^th^
	The future of children	0.81	0.65	Sig.	2^th^
	The future wife	0.75	0.56	Sig.	3^rd^
	Concern	0.66	0.44	Sig.	6^th^

After conducting two-steps confirmatory factor analysis, we deal with examining the adequacy of fitting model. It is worth nothing that among different indices of the propriety model such aschi-square indices, Root Mean Square Error of Approximation (RMSEA), Normed Fit Index (NFI), Non-Normed Fit Index (NNFI), Root Mean Square Residual (RMR), Goodness of Fit Index (GFI), Adjusted Goodness of Fit Index (AGFI) and Incremental Fit Index (IFI), these indices can determine the Goodness of fit statistics.

Table 5 shows that the fitting indices of the analysis pattern in CLDQquestionnaire.

**Table 5:** Indicates of CLDQmodel

Indicates	Recommended value	Scores	Fitness
Chi-square	-	234.20	
P-value	-	0.0000	
d.f	*df* ≥ 0	147	acceptable
χ^2^/*df*	χ^2^/*df* < 3	1.59	acceptable
RMSEA	RMSEA < 0.1	0.053	acceptable
NNFI	NNFI> 0.8	0.94	acceptable
NFI	NFI > 0.8	0.89	acceptable
AGFI	AGFI> 0.8	0.86	acceptable
GFI	GFI> 0.8	0.89	acceptable
CFI	CFI > 0.8	0.95	acceptable
IFI	IFI > 0.8	0.95	acceptable
RMR	Coverage to zero	0.067	acceptable

As you see in Tables 5 and 6, the value of the chi-square statistic in CLDQ model is 234.20. Also the degree of freedom of the model equals 147 that the sum of their ratio equals 1.59 and in the general health model the value of the chi-square statistic is 734.73, the degree of freedom of the model also equals 248 that the sum of their ratio equals 2.96 that is in the range of acceptable value. On the one hand, fitting indices of the model such as NNFI, NFI, AGFI, GFI, CFI and IFI are all acceptable and satisfactory, on the other hand RMSEA index for CLDQ model equals 0.053 which is less than 0.1 and RMR index is also 0.067 and for general health model equals 0.070 and 0.079, respectively which is a small amount. It shows that the resulting model have a very good adaptation indices and demonstrates the suitability of the research model.

**Table 6:** Indicates of general life quality model

Indicates	Recommended value	Scores	Fitness
Chi-square	-	734.73	
P-value	-	0.0000	
d.f	*df* ≥ 0	248	acceptable
χ^2^/*df*	χ^2^/*df* < 3	2.96	acceptable
RMSEA	RMSEA < 0.1	0.070	acceptable
NNFI	NNFI> 0.8	0.95	acceptable
NFI	NFI > 0.8	0.92	acceptable
AGFI	AGFI> 0.8	0.80	acceptable
GFI	GFI> 0.8	0.84	acceptable
CFI	CFI > 0.8	0.96	acceptable
IFI	IFI > 0.8	0.96	acceptable
RMR	Coverage to zero	0.079	acceptable

**Diagram 1:**
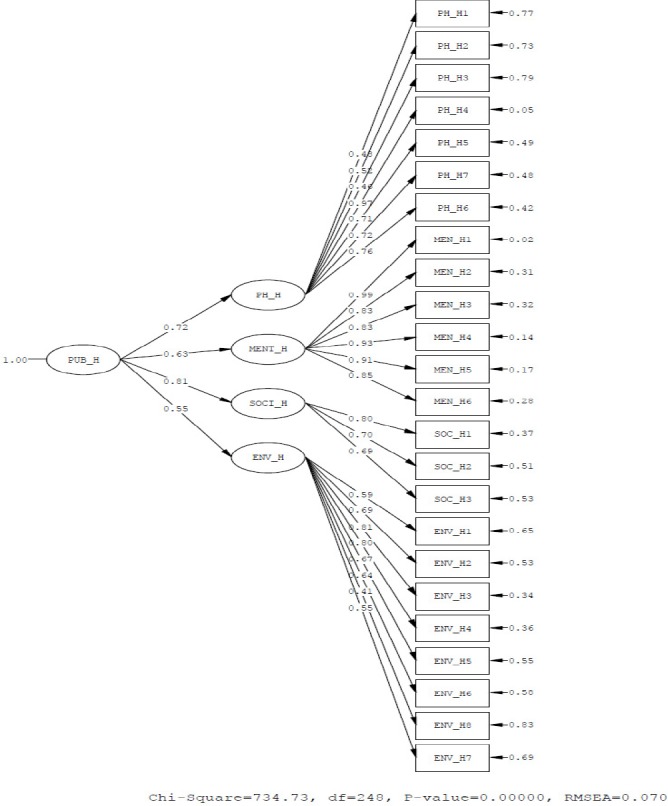
The fitting indices of general life quality model

**Diagram 2:**
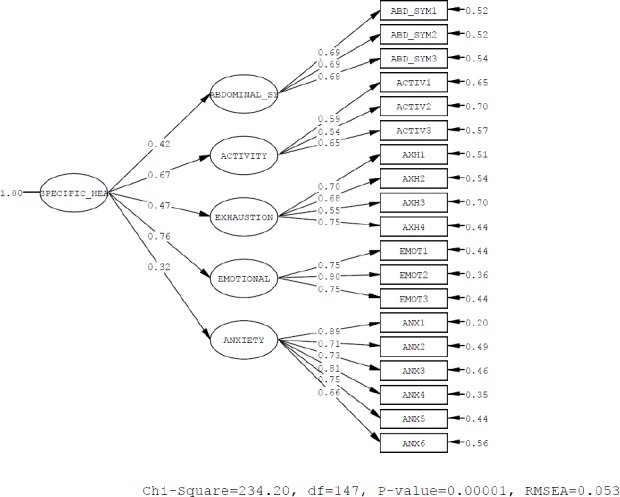
The fitting indices of CLDQ model

## 4. Discussion

In this study, the all variables in general life quality was less than average. Also, social relationship domain had the highest coefficient of effectiveness on general life quality and physical health, mental health and environmental health had the lowest score, respectively. In assessment of quality of life in hepatitis B patients compared with healthy people, physical health criteria had highest score and environmental health had lowest score (Abedi & Rostami, 2012), but the result study of Ghanbarishowed that mental and physical health had the lowest effect on life quality (Ghanbari, Farmanbar, & Mansourghanaii, 2010). Our study found that the emotional function of the quality of life in hepatitis B patients had the most coefficients of effectiveness and activity, fatigue, abdominal symptoms and worry dimensions had the lowest score, respectively, in making CLDQ domains of hepatitis B patients. CLDQ scores in Zhuang et al. study showed that CHB group among three patient groups scored the highest on overall score and all domains except WO, significantly different from any of two other groups on overall score and half of the domains (Zhuang et al., 2014) that this result is the same as our study; WO had the lowest factor loading in CLDQ scores. Bernstin believes that attention to life quality is the main concerns of chronic patients and stated that patents care should propel to maintain life quality such as the ability of maintain job, the ability to maintain a relationship with friends, wife and children, the ability of continued happiness and enjoyment of pleasant situation (Bernstein, 2006). In current study, relationship with other members of family in social relationship domain had the highest factor loading and in emotional health domain, items such as enjoy life and satisfy with yourself were the important items in patients.

Performed studies on 642 patients with chronic hepatitis Bshowed that the scores of life quality in patients in comparison with the control group were lower than healthy people. HCV infection increase fatigue, decrease function ability of work, home and the school and patients don’t have any self- confidence and always are worries about their health situation in the future (Jianqian, 2013). Assessment of quality of life in cancer patientsshowed the General life quality in patients was weak and general life quality was lower than particular quality of life (Farzianpour, Shojaee, Abedi, & Rostami, 2014) that this is in accordance with our study. In current study variables of general quality of life had been below average. In some studies on quality of life, the effective variables were individual features (Ware, Bayliss, Mannocchia, & Davis, 1999), but in this study, appearance variable in emotional health domains was the 3^th^ priority between other items. Depressive disorders were associated with worse scores in overall health relatedquality of life and in all domains (Bernstein, 2006) but in current study, anxiety and depression had the 4^th^ priority in emotional health dimension. Fatigue was associated with lower scores in physical and psychological domains, and married status with higher scores in psychological health related quality of life and there wasstrong correlation among scores of depression; fatigue and health related quality of life (Bernstein, 2006). Also in this study in fatigue dimension, feeling tired in sport, fatigue during the day, walking had the highest priority to lowest priority items.

The life quality concept and more especially, healthrelated quality of life, implies physical, emotional and social health, i.e. it defines the issues affected by patients’ experiments, their expectancies or beliefs and understandings (Heidarzadeh et al., 2007). So, social relationship and emotional function must be properly investigated and managed in hepatitis B patients in order to improve life quality. Generally, it seems that deduction in life quality could result from problems in social relationship and physical health in general and also emotional function and activity in hepatitis B patients. WHOQOL-BREF and CLQD proved to be a useful instrument to assess general life quality in patients and can be helpful to find practical strategies to improving life quality in these patients. So, managers should be aware about promotion of life quality by practical program and intervention with every group to prepare the appropriate level of life quality. The main steps for improving the quality of life can be fully integrated of the care program of these patients in network system, easy access and facilitating in intervention to improve the life quality is offered.
